# Enhancing the Performance of Triboelectric Generator: A Novel Approach Using Solid–Liquid Interface-Treated Foam and Metal Contacts

**DOI:** 10.3390/polym15102392

**Published:** 2023-05-20

**Authors:** Quang Tan Nguyen, Duy Linh Vu, Chau Duy Le, Kyoung Kwan Ahn

**Affiliations:** 1 Graduate School of Mechanical Engineering, University of Ulsan, 93, Daehak-ro, Nam-gu, Ulsan 44610, Republic of Korea; 2 School of Mechanical Engineering, University of Ulsan, 93, Daehak-ro, Nam-gu, Ulsan 44610, Republic of Korea

**Keywords:** direct-current triboelectric generators, solid–liquid interface-treated foam, material work function, mechanical-to-electrical conversion, self-powered sensor

## Abstract

This work introduces a novel approach for enhancing the performance of a triboelectric generator (TEG) by using a solid–liquid interface-treated foam (SLITF) as its active layer, combined with two metal contacts of different work functions. SLITF is made by absorbing water into a cellulose foam, which enables charges generated by friction energy during the sliding motion to be separated and transferred through the conductive path formed by the hydrogen-bonded network of water molecules. Unlike traditional TEGs, the SLITF-TEG demonstrates an impressive current density of 3.57 A/m^2^ and can harvest electric power up to 0.174 W/m^2^ with an induced voltage of approximately 0.55 V. The device generates a direct current in the external circuit, eliminating the limitations of low current density and alternating current found in traditional TEGs. By connecting six-unit cells of SLITF-TEG in series and parallel, the peak voltage and current can be increased up to 3.2 V and 12.5 mA, respectively. Furthermore, the SLITF-TEG has the potential to serve as a self-powered vibration sensor with high accuracy (*R*^2^ = 0.99). The findings demonstrate the significant potential of the SLITF-TEG approach for efficiently harvesting low-frequency mechanical energy from the natural environment, with broad implications for a range of applications.

## 1. Introduction

The demand for alternative renewable energies and energy harvesting technologies has increased in recent years, prompting scientists to search for new, green, reliable, and cost-effective sources of energy. While mechanical energies from wind, water flow, and ocean waves have been established as important sources of power generation, small mechanical energies such as human motions, low-frequency vibration, and raindrops have been overlooked due to their low input energy [1]. However, the rise of portable and functional electronic devices, such as those used in the Internet of Things (IoT), has created a need for continuous, stable, and portable power supplies, making energy harvesting techniques a viable option for powering such devices [2,3,4]. Additionally, water-based energy harvesting technology has garnered significant research interest due to its potential, environmental friendliness, and wide availability [5,6,7]. As a result, researchers are focusing on developing devices capable of converting mechanical energy into electrical energy in a high-humidity environment. 

The triboelectric generator (TEG) is a technology that can effectively harvest energy from small mechanical energy sources, introduced by Wang et al. [8,9,10]. Traditional TEGs work by coupling contact electrification (CE) and electrostatic induction, which involves the accumulation of charges with different signs on the corresponding surfaces when two materials are contacted or slid against each other [11]. Up to now, various TEG designs have been developed; however, most generally exhibit alternating current (AC) outputs, and a rectification method is required for converting to direct current (DC) before using to power electronic devices. This can be inconvenient and inefficient, particularly for low-power applications. To address this issue, novel strategies/technologies are being developed to directly convert mechanical energy into DC power, with a focus on enhancing the output power density, particularly the current density [12,13,14].

Recently, several methods have been proposed for generating DC power using water-based systems. These methods include DC-TEGs, which use water electrification and phase control with an array of disks [15], gas–liquid two-phase flow-based TENG devices that combine contact electrification and the breakdown effect [16], and droplet-based electricity generators that produce DC power through direct charge transfer at the water–metal contact interface [17,18,19]. Additionally, researchers have developed devices that convert mechanical energy from the movement of a water droplet in any direction within a layered structure composed of graphene, water, and a semiconductor [20], as well as the movement of water between two semiconductors [21]. A particularly interesting development is the dynamic junction theory proposed by Solares-Bocmon et al. [22]. This theory explains the mechanism of the direct current generation at solid–liquid interfaces, which has important implications for the design and optimization of water-based energy harvesting devices. According to this theory, when two materials with different work functions are brought into contact, a charging effect occurs that aligns their Fermi levels and develops a built-in voltage to prevent further net charge transfer. When there is a relative mechanical movement at the contact interface, extra electrons and holes are generated due to the dynamic junction. These are then separated by the built-in electric field, producing direct current in the external circuit. This means that DC power can be generated due to the movement of the water between two conductors. Besides, to solve the problem of insufficient generated current, various approaches have been conducted, such as the electrospinning technique [23,24,25,26]. By improving the conductivity of the triboelectric layer, the electron transport capacity can be improved, resulting in enhancing the output current density of the TEGs [27].

On another hand, the output performance of TEGs has been reported to generally decrease in the presence of water due to charge dissipation of the triboelectric charges, which can limit their effectiveness [28,29,30]. To overcome this limitation, new materials with high triboelectric charges in high-humidity environments need to be identified. For instance, Wang et al. [31] developed a TENG that utilizes the participation of water molecules fixed by hydrogen bonds formed with hydroxyl groups in a polyvinyl alcohol film to enhance the output performance. This is achieved by increasing the charge quantity and, subsequently, the triboelectricity. Mandal et al. [32] proposed a DC generator that employs an active protein layer with a hydrogen-bonded network of water molecules to transfer charges and generate electricity between two dissimilar metal contacts. Interstingly, cellulose has emerged as a promising functional material for the development of low-cost and eco-friendly energy harvesting technologies [27]. Cellulose contains abundant hydroxyl groups that endow it with a strong electron donation capacity, making it highly suitable for the triboelectric effect [33,34]. Moreover, cellulose is hydrophilic and tends to strongly interact with water. When cellulose comes into contact with water, the hydroxyl groups spontaneously form hydrogen bonds with water molecules. This leads to the fixing of water molecules on the surface of cellulose and the formation of a conductive path through the hydrogen-bonded network of water molecules [35,36,37,38]. This property of cellulose could be an important factor to explore in solving the problem of the decreasing trend of electricity performance in high-humidity environments.

In this paper, we introduce a novel direct-current triboelectric generator (TEG) that uses a solid–liquid interface-treated foam (SLITF), made by absorbing water into a cellulose foam, as its active layer to generate electric power from low-frequency vibration energy. The mechanism of this device relates to the CE and the charging effect between two materials with different work functions, a well-known phenomenon that was thoroughly characterized by Lord Kelvin [39,40]. This device is capable to convert mechanical energy from the relative sliding of the SLITF into DC power based on the dynamic junction of the SLITF and metal contacts. The study systematically examined the impacts of working parameters and material selection on the SLITF-TEG ‘s output performance. Our findings showed that SLITF-TEG can produce an impressive current density of 3.57 A/m^2^, a power density of 0.174 W/m^2^, and an induced voltage of approximately 0.55 V. The voltage and current can be increased up to 3.2 V and 12.5 mA by simply synchronizing the outputs of six-unit cells of SLITF-TEG connecting in series and parallel, respectively. Six-unit cells in series can produced an output energy of 2 mJ in 100 s and store it directly in a 1 mF capacitor without requiring rectification. This amount of energy is adequate for powering various electronic devices. Additionally, we demonstrate the great accuracy and potential application of SLITF-TEG for measuring the vibration frequency/sliding velocity with a high coefficient of determination (*R*^2^ = 0.99). Overall, these results illustrate the high potential of SLITF-TEG in mechanical energy harvesting and the field of self-powered sensor fabrication.

## 2. Materials and Methods

### 2.1. Materials

For preparing the materials, aluminum (Al) and copper (Cu) tapes were bought from Ducksung Hitech (Ducksung Hitech Co., Ltd., Seoul, South Korea). The cellulose foam is manufactured by Hankook Tamina (Hankook Tamina Co., Ltd., Hanam, South Korea). Distilled water was used from our research laboratory. Indium tin oxide (ITO) electrode (10 Ω/sq), polytetrafluoroethylene film (PTFE, 100 μm-thick), and polyvinylidene fluoride (PVDF, 50 μm thick) were purchased from Sigma-Aldrich (Sigma-Aldrich, St. Louis, MO, USA). Besides, the mica, nylon, and resistors were purchased from a local market. Capacitors were purchased from Rubycon (Rubycon Corporation, Nagato, Japan).

### 2.2. Fabrication of the SLITF-TEG

To fabricate the SLITF-TEG, the Cu and Al tapes were cut into rectangular pieces of the same size (2 cm × 4 cm or 2 cm × 3.5 cm), and devised the electrical circuit as the bottom and top electrodes, respectively. A PTFE film (8 cm × 8 cm) was utilized as the dielectric layer and a solid–liquid interface-treated foam (SLITF), fabricated by absorbing distilled water (1 to 3.5 mL) into a cellulose foam (3 cm × 4 cm or 2 cm × 3.5 cm; 0.5 cm-thick), was used as an active friction layer. The Cu electrode was mounted on the PTFE layer, whereas the Al electrode was laminated to the SLITF and fixed to a movable substrate. The SLITF reciprocates on the surface of the dielectric layer and the bottom electrode, resulting in the lateral sliding mode of operation of the SLITF-TEG. Load resistances (10 Ω to 10 MΩ) and capacitances (0.1 to 6.8 mF) are applied in the external circuit.

### 2.3. Measurement and Characterizations

To assess the characteristics of the SLITF-TEG, the voltage and current were measured by a Digit Graphical Sampling Multimeter model DMM7510 of Keithley Instruments, Inc. (Cleveland, OH, USA). The surface morphology was investigated using Field Emission Scanning Electron Microscopes (FE-SEM, JSM-7600F, JEOL, Tokyo, Japan), whereas the contact angle was measured by SmartDrop (Femtofab Co., Ltd., Seongnam, Korea) to evaluate the hydrophilicity of the material. In addition, the elemental composition and chemical states of cellulose were determined by Thermal Scientific Nicolet iS5 FT-IR (Fourier-transform infrared spectroscopy) Spectrometer (Thermal Fisher Scientific Inc., Madison, WI, USA). The crystal structure of the cellulose foam was analyzed by an X-ray Diffractometer system (D/MAX-25000V, Rigaku, Tokyo, Japan) using monochromatic CuKα radiation with the wavelength λ = 1.54178 Å.

### 2.4. Method

The general physical process for energy conversion has three important steps: charge generation, charge separation, and charge flow [41]. In the proposed mechanism, charges are generated by friction energy during the relative sliding of the SLITF and separated under the effect of the built-in electric field developed between the two metal contacts. These charges are facilitated to transfer through metal/SLITF/metal counterparts via a conductive path formed inside the SLITF in one orientation, leading to generating a direct current through an external circuit. The impacts of working parameters and material selection on the output performance of the SLITG-TEG are systematically investigated, including vibration frequency, water absorption, different materials of the dielectric layers, and electrode pair materials.

## 3. Results and Discussion

### 3.1. Characterization of the Cellulose Foam

**Figure 1:**
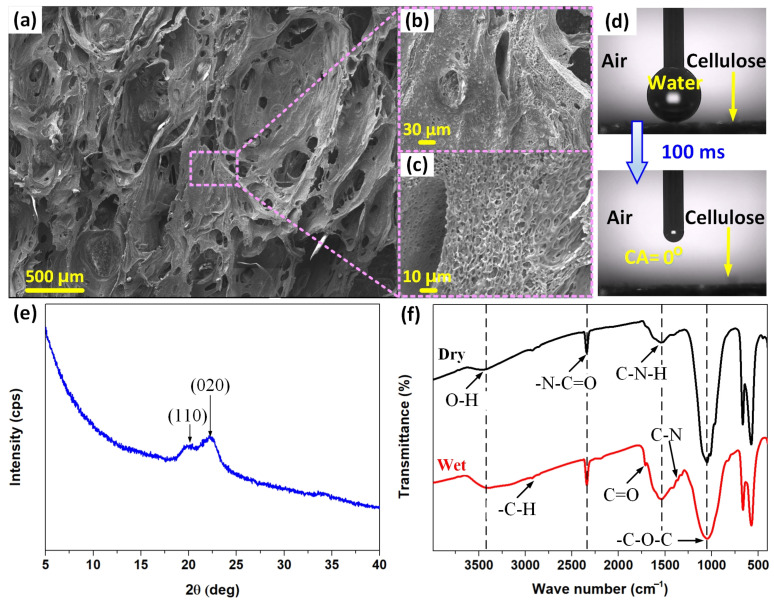
FE-SEM images of cellulose foam showing (**a**) macropores, (**b**) micropores on the wall of macropores, and (**c**) nanopores on the wall of micropores. (**d**) Water wettability of the cellulose foam in air. (**e**) XRD patterns of cellulose foam. (**f**) FT-IR spectra of dry and wet cellulose foam.

Polymers containing nitrogen and oxygen with pyridine amide, amine, or hydroxyl groups develop the most positive charges [11]. Thus, cellulose, which contains numerous hydroxyl groups with a strong electron donation capacity, is a high electropositivity material for triboelectrification. In addition, the hydrogen-bonded network formed due to the interaction between water molecules and the hydroxyl groups of cellulose creates a conductive path [35,36,37,38] that can facilitate charge transfer during the electricity generation process. The characterization of the cellulose foam is presented in Figure 1.

The morphology of the cellulose foam was characterized by FE-SEM and the results are reported in Figure 1a–c. The FE-SEM images show a 3D porous network structure with interconnected pores consisting of numerous macropores randomly distributed on the surface that are connected to form an open channel system (Figure 1a). The foam-like morphology can act as water channels that can take up high quantities of water [42]. Especially, it can be observed a secondary structure with micropores in the cell wall of the macropores on a length scale on the order of a few microns (Figure 1b) and a third-order with super-porous structures with dimensions of up to 1 micrometer (Figure 1c). This morphology increases the microporosity of the network, thus leading to a higher absorption capacity of the cellulose foam [43]. Figure 1d demonstrated the super-hydrophilicity of the cellulose foam, where a water droplet (3 μL) immediately spreads out and is absorbed by the cellulose foam within 100 ms upon contacting the surface of the cellulose foam, resulting in a water contact angle of nearly zero.

The XRD pattern of the cellulose foam is depicted in Figure 1e. A CuKα X-ray source with a voltage of 45 kV and a current of 45 mA was used to record the diffraction patterns. The main diffraction peaks appear at 2*θ* = 20° and 22.1°, corresponding to the (110) and (020) crystal planes, respectively, which are supposed to represent the typical cellulose II crystal structure [44,45]. The peaks corresponding to (110) and (020) are formed by inter- and intra-hydrogen bonding [46], proposing a hydrogen-bonded network in cellulose [47].

Additionally, the elemental composition and chemical states of cellulose were analyzed by using FT-IR spectroscopy, as summarized in Figure 1f. The intermolecular hydrogen bonding in cellulose, which relates to the surface -OH participation of hydrogen bonds, is generally shown at a wavenumber of 3447 cm^−1^ [47]. The peak at 1550 cm^−1^ is attributed to the C-N-H of the amine group (the stretching vibration of C-N and the bending vibration of N-H) [48,49]. Other peaks observed in the FT-IR include -C-H bonds in polysaccharides (at 2914 cm^−1^), -N=C=O stretching (at 2340 cm^−1^), C=O stretching (at 1730 cm^−1^), C-H deformation vibration (at 1375 cm^−1^), -OH in-plane bending (at 1330 cm^−1^), C-N stretching vibration (at 1321 cm^−1^), and -C-O-C pyranose ring vibration (at 1050 cm^−1^) [50,51,52,53]. 

### 3.2. Electric Output Characteristics and Working Mechanism of the SLITF-TEG

The experimental setup includes a slider crank mechanism that simulates mechanical vibration, a SLITF-TEG cell, and external measuring equipment, as shown in Supplementary Material Figure S1. For constructing the SLITF-TEG cell, we used copper (Cu) and aluminum (Al) films (2 cm × 4 cm) as the bottom and top electrodes, respectively, and placed a PTFE film (8 cm × 8 cm) as the dielectric layer. We then fabricated the SLITF by absorbing 3.5 mL of distilled water into a cellulose foam (3 cm × 4 cm × 0.5 cm, 0.64 g-mass). The Cu electrode was mounted on the PTFE layer, whereas the Al electrode was laminated to the SLITF and fixed to a movable substrate (8.78 g mass). 

**Figure 2:**
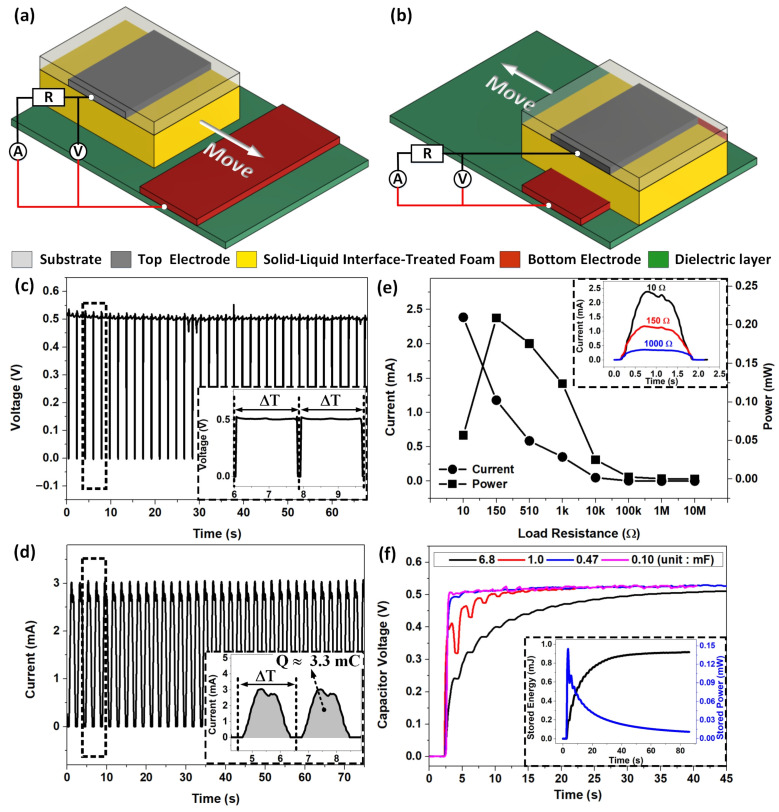
The electrical characteristics of the SLITF-TEG. 3D schematic illustrations of the measurement setup and the external circuit corresponding to (**a**) disconnected and (**b**) connected states of the ideal cycle of operation. (**c**) The open-circuit voltage. Inset image: partial magnified view of the voltage. (**d**) The short-circuit current. Inset image: partial magnified view of the current signal and estimated charge transferred. (**e**) The impedance-matching curve of the SLITF-TEG. Power and average current as a function of load resistance. Inset image: Enlarged view of typical peaks of current output at different load resistances. (**f**) Charging voltage on various load capacitances. Inset image: Stored energy-time and stored power-time relationships for a fixed capacitor of 6.8 mF.

To evaluate the electric output characteristics of SLITF-TEG, The SLITF was driven to reciprocate on Cu and PTFE surfaces with a period time of about 2 s, corresponding to a sliding velocity of 3.5 cm/s, as shown in Figure 2a,b. Then, we measured the voltage output under the open-circuit condition and plotted the results in Figure 2c. It can be observed that the device can produce a continuous DC voltage (*Voc*) with all positive pulses due to the continuous reciprocating motion, with an average peak value of ~0.52 V and a time interval of the pulse ∆*T* of ~2 s. We also measured the current output under the short-circuit condition (*Isc*). The current peaks showed an increasing trend with the increasing number of movement cycles and approach a stable saturation value after a sufficiently long time, as shown in Supplementary Material Figure S2a. Figure 2d displays the *Isc* curve based on the saturation, in which a similar signal waveform to the voltage output is observed. From this figure, the peak current value can reach ~3.12 mA, corresponding to a current density of 2.6 A/m^2^, and the time interval between current peaks is also ∆*T*. The estimated charge transfer during one movement cycle obtained 3.3 mC, corresponding to a charge density of 2.75 C/m^2^, as shown in the inset image. The estimated charge transfer *Q* is described by Equation (1)

(1) Q=∫i.dt

where *i* is the instantaneous current and *t* is the time.

Importantly, we observed that the DC pulse outputs were obtained when the SLITF is brought into contact with the Cu electrode following the reciprocating motion. Otherwise, both voltage and current outputs are negligible, and the current flow is unidirectional from the Cu electrode to the Al electrode (Supplementary Material Videos S1 and S2). We also confirmed experimentally the DC characteristics of electric outputs by applying various load resistances and capacitances in the external circuit. The current outputs measured at 10 Ω, 150 Ω, and 1000 Ω load resistances are plotted in Supplementary Material Figure S2b, and the corresponding enlarged views of typical peaks are illustrated in the inset image of Figure 2e. All these results illustrate pulsed DC characteristics of SLITF-TEG. 

We also investigated the dependence of current output on the load resistance in a range from 10 Ω to 10 MΩ and found that the value of peak current can reach 2.386 mA at 10 Ω and dramatically decreases to 50 μA and then 50 nA when the load resistance increases to 10 kΩ and then 10 MΩ, respectively. Figure 2e also displays the output power (P) as a function of external resistance. The power initially increases with increasing the external resistance to reach a maximum value of 208.86 μW at 150 Ω, corresponding to a power density of 0.174 W/m^2^, and then decreases with a further increase in the *R_L_*-value. We calculated the output power by using Equation (2)

(2) $$P=R_{L}\cdot i^{2}$$

where *i* is the peak current at the corresponding load resistance (*R_L_*).

The ability of the SLITF-TEG to generate DC outputs allows for the direct charging of energy storage units without the need for a rectifier. We verified this capability by exploring the charging behaviors across different load capacitances *C_L_* (0.1 to 6.8 mF) of the SLITF-TEG, and the experimental results are presented in Figure 2f. The trends of charging voltage with the charging time and various load capacitances *C_L_* have similar tendencies, where the capacitor is typically charged rapidly in the beginning and slows down until reaching a saturated voltage (Supplementary Material Video S3). It is noted that the smaller *C_L_* needs less time to reach saturation than the larger *C_L_*. Moreover, the stored power–time relationships for a fixed capacitor *C_L_* = 6.8 mF are plotted in the inset image, which indicates an optimum charging time where the maximum stored power is obtained. This inset image also shows the stored energy–time relationship, which increases gradually until reaching a saturation value of approximately 0.884 mJ. We calculated the stored energy *Ws* by using Equation (3)

(3) $$Ws=CV^{2}/2$$

where *V* is the voltage and *C* is the capacitance of the capacitor.

**Figure 3:**
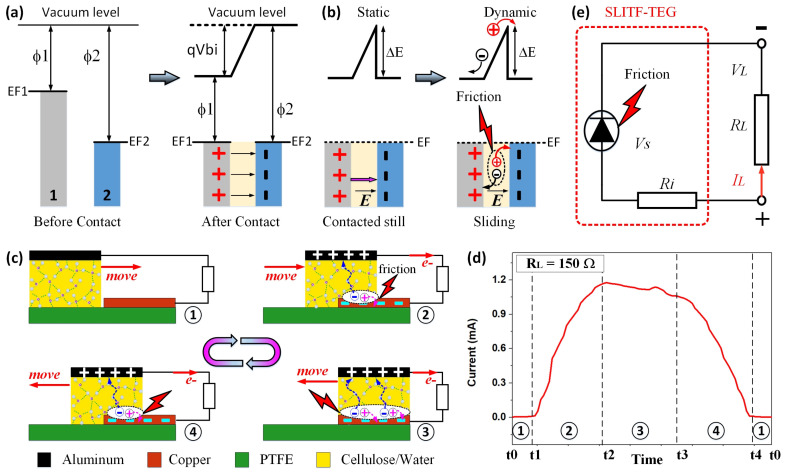
(**a**) Electron energy levels of materials 1 and 2 before and after making contact. Here, EF1 and EF2 are Fermi levels of materials 1 and 2, respectively. (**b**) Space charge, built-in electric field, and potential after two materials are in contact at a static state and separation charges in a dynamic state. (**c**) The working mechanism of the sliding mode SLITF-TEG. (**d**) Magnified view of a current output signal during an ideal operating cycle of the SLITF-TEG. (**e**) Equivalent circuit diagram of the SLITF-TEG.

The SLITF-TEG works by exploiting the natural electron transfer that occurs when two materials with different work functions come into contact, in which electrons transfer from the material with a lower work function to the material with a higher work function. This transfer continues until the Fermi levels of both materials align, resulting in a built-in electric field (*E_bi_*) and a voltage (*V_bi_*) across the materials (Figure 3a). The built-in voltage $V_{bi}$ is given by Equation (4)

(4) $$V_{bi}=(\phi 2-\phi 1)/q$$

where *ϕ*1 and *ϕ*2 are the material work functions of materials 1 and 2, respectively, and *q* is the elementary charge [22,39,40,54]. When the two materials are in static contact (i.e., contacted still), the equilibrium state is established, and the built-in electric field and voltage prevent further net charge transfer. On the contrary, when they are in dynamic contact (i.e., sliding), extra charges (or nonequilibrium carriers) can be generated due to mechanical friction energy (Figure 3b). These extra charges (*Q_tri_*) can come from two sources: (1) charges generated by the CE during the sliding friction and (2) charges generated by energy released as a result of bond breakage at the interface during dynamic friction. These extra charges will be separated under the effect of the built-in electric field in one orientation, providing the main driving force of DC generation in the SLITF-TEG. The generated current density ($J_{SC}$) can be expressed by Equation (5)

(5) $$J_{SC}=Q_{tri}\cdot \mu \cdot E_{bi}$$

where *μ* is the mobility of materials [55,56]. Therefore, the use of SLITF plays a crucial role in generating high-output performance. As mentioned above, the interaction between the water and cellulose leads to the fixing of water molecules on the surface of cellulose and the formation of a conductive path through the hydrogen-bonded network of water molecules inside the cellulose foam. Accordingly, more charges will be generated during the friction and these charges will be facilitated to flow through the whole circuit, leading to generating high-output performance, particularly high-output current. 

The working mechanism of SLITF-TEG is illustrated in Figure 3c. When the SLITF was not in contact with the bottom electrode (Cu), there was no current output in the external circuit (Figure 3c, step 1). However, when it was brought into contact, the top electrode (Al) affixed to SLITF was connected electrically with the Cu electrode through a conductive path formed by the hydrogen-bonded network of water molecules inside SLITF (Figure 3c, step 2). This leads to the development of the built-in electric field/voltage across Al and Cu electrodes caused by their difference in work functions. During the sliding of SLITF on the Cu electrode, extra charges are generated due to CE at the contact interface and then separated by the built-in electric field via the conductive path. The work functions of Cu and Al were approximately 4.7 eV and 4.2 eV [22,32,57,58,59], respectively; thus, the built-in electric field points from the Al electrode to the Cu electrode. As a result, charges will flow through SLITF/metals interfaces to the external circuit in one orientation. Concisely, negative charges/electrons generated by the friction energy transfer from the Cu electrode to SLITF and then to the Al electrode via the conductive path formed inside SLITF under the effect of the built-in electric field and eventually to the Cu electrode via the external circuit. The output electrons flowed unidirectionally from the Al electrode to the Cu electrode, resulting in DC power generation. The DC outputs are still obtained even when SLITF continues sliding to the end of the right alignment (Figure 3c, step 3) and then moving back to the initial state (Figure 3c, step 4). The reverse lateral sliding process is the same as in Figure 3c, step 2. Thereafter, no current flowed in the external circuit when SLITF separated from the Cu electrode and moved backward to the initial position as in Figure 3c, step 1.

Briefly, the working mechanism of SLITF-TEG can be divided into two primary states. In the first state, the SLITF and the Cu electrode were separated, and the electric outputs were negligible (Figure 3c, step 1). In the second state, the SLITF was brought into contact and slid on the surface of the Cu electrode (Figure 3c, steps 2–4), and voltage/current was generated. The maximum output was achieved when the SLITF and the Cu electrode are in full contact (Figure 3c, step 3). Consequently, the SLITF-TEG can produce continuous pulsed DC electricity by periodically sliding the SLITF. Notably, the DC characteristics of the device depend on the built-in electric field developed between two electrodes. 

A detailed current output signal during one movement cycle, measured at a load resistance of 150 Ω, is shown in Figure 3d and Supplementary Material Figure S3a, demonstrating the consistency between the electrical responses and the working mechanism. The current output starts when the SLITF contacts the Cu electrode (*t*1) and increases until the SLITF and the Cu electrode are in full contact (*t*2). When the SLITF moves backward (*t*3), the current value decreases as the contact area between the SLITF and the Cu electrode decreases. Once the SLITF separates from the Cu electrode (*t*4), the current becomes negligible. The corresponding experiment is presented in Supplementary Material Figure S3b.

It is worth noting that the Cu/SLITF/Al structure acts as a diode-like component, allowing current to flow through the structure in a single orientation during the electricity generation process. Looking at the device from a circuit perspective, the SLITF-TEG can be modeled as a friction-induced generation diode-like voltage source (*Vs*) (Cu/SLITF/Al—related to the voltage difference formed by the nonequilibrium carriers during the sliding friction), an internal resistance *Ri* (SLITF), and a load resistance *R_L_*. This device can produce DC output (*I_L_*) and voltage (*V_L_*) through the external resistance *R_L_*. Figure 3e illustrates the equivalent circuit diagram of SLITF-TEG. 

### 3.3. Working Parameter Responses of the SLITF-TEG

In order to gain further insights into the electrical output and impedance characteristics of the SLITF-TEG, we explored the influence of different working parameters. As a model study, we first investigated the important role of mechanical friction in generating high-output performance. We measured the electric responses when keeping the SLITF stationary on the Cu electrode and then compared it with that of the sliding mode mentioned earlier. The electric outputs of the stationary mode showed a constant voltage of approximately 0.52 V, while the current dramatically decreased from 75 μA to a stable saturation value of 8 μA after 700 s (Supplementary Material Figure S4). Although the maximum voltages in both stationary and sliding modes were almost the same, the current generated in the sliding mode was more than 375 times higher than those in the stationary mode, demonstrating the essential role of mechanical friction and the CE in enhancing the output performance of the SLITF-TEG.

**Figure 4:**
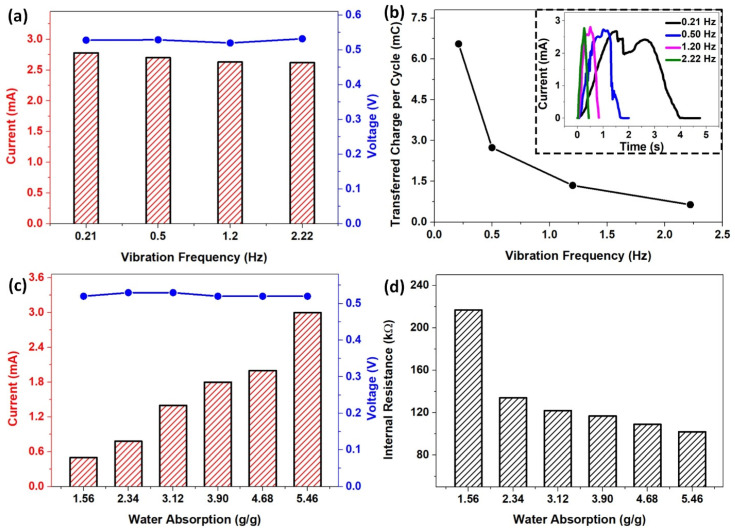
The dependence of (**a**) open-circuit voltage and short-circuit current and (**b**) Transferred charges during one movement cycle on the vibration frequency. Inset image: Enlarged view of typical peaks of current output with different frequencies. The dependence of (**c**) open-circuit voltage and short-circuit current and (**d**) Internal resistance of SLITF on the water absorption.

Simultaneously, we examined the effects of sliding velocity, which is driven by the vibration frequency of the reciprocating motion, on the maximum instantaneous electric outputs (*Voc* and *Isc*). By comparing the electric outputs under different vibration frequencies in the range from 0.21 to 2.22 Hz, we found that the sliding velocity had little effect on the peak values of *Voc* and *Isc*, with a slight difference of less than 1% and 6%, respectively (Supplementary Material Figure S5 and Figure 4a). However, we observed a decreasing trend in the time interval for reaching the maximum instantaneous current and the transferred charge (*Qc*) during one movement cycle with increasing vibration frequency, as depicted in Figure 4b. From this figure, the value of *Qc* decreased from 6.55 to 0.64 mC/cycle when the vibration frequency increased from 0.21 to 2.22 Hz. This could be due to the decrease in the working period caused by the increase in the vibration frequency.

We also investigated the essential role of water absorption of SLITF on the electric outputs to further optimize the output performance of the SLITF-TEG. Water absorption is defined as the amount of water absorbed by the cellulose foam and is calculated as the ratio of the weight of water absorbed to the weight of the dry cellulose foam. The results showed an impressive power generation capacity, in which the current output exhibited an upward trend from 0.5 to 3.12 mA, corresponding to a current density from ~0.42 to 2.6 A/m^2^, when the water absorption of the SLITF increased from 1.56 to 5.46 g/g, as presented in Figure 4c. This was presumably due to the effect of the internal resistance of SLITF on the current output. Previous studies have demonstrated that the increasing amount of absorbed water leads to an increase in the concentration of hydrogen bonds, resulting in a decrease the internal resistance [32]. Therefore, charges generated during the sliding motion are facilitated to transfer through the conductive path formed by the hydrogen-bonded network of water molecules inside SLITF, leading to generating high output current. To test this hypothesis, we measured the internal resistance of SLITF at different water absorption levels, finding that the internal resistance of SLITF considerably decreased from 217 to 102 kΩ when the water absorption increased from 1.56 to 5.46 g/g (Figure 4d). Meanwhile, the peak voltage output remained almost the same at ~0.52 V despite the increase in water absorption.

Subsequently, to demonstrate the crucial role of the SLIFT (cellulose foam treated by water) in the enhancement of energy conversion of the SLITF-TEG, we compared it to a conventional solid-solid triboelectric generator (SS-TEG) with the same structural design but using dry cellulose foam instead of SLITF. We found that SS-TEG exhibited a peak *Voc* of about 0.2 V and *Isc* of 30 nA with AC output characteristics (Supplementary Material Figure S6). Furthermore, we also realized an electrochemical cell (EC) composed of Al and Cu electrodes immersed in water, which implied constant voltage and current outputs with average values of ~0.5 V and 5 μA, respectively (Supplementary Material Figure S7a). Comparison between the SLITF-TEG, SS-TEG, and EC found significant improvements in generating high output current of SLITF-TEG, with over 600 times more output current than both the SS-TEG and EC (Supplementary Material Figure S7b). Notably, the internal resistances in the cases of SS-TEG and EC are respectively obtained at 6 MΩ, and 1.2 MΩ, considerably higher than the internal resistance of the SLITF-TEG (102~207 kΩ). Overall, these results strongly support our conclusion that fabricated SLITF is essential for an effective mechanical-to-electrical energy conversion technology.

**Figure 5:**
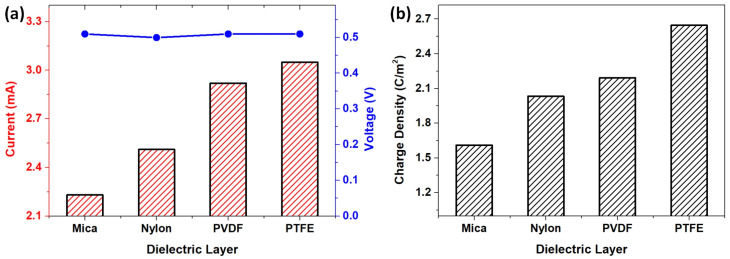
The dependence of (**a**) open-circuit voltage and short-circuit current and (**b**) transferred charge density during one movement cycle using different polymers for dielectric layers.

Further research revealed how the dielectric layer impacts the output performance. In the triboelectric mechanism, selecting material pairs with a large difference in surface charge is critical for generating more triboelectric charges and then maximizing the outputs. As previously mentioned, water and cellulose foam are combined into one reactive entity and participate in CE as a high electropositivity material. Certainly, a dielectric layer with a high negative surface charge is suitable for generating a relatively high electrical [60]. To confirm this, we considered different polymers within the triboelectric series, including (most negative) PTFE, PVDF, nylon, and mica (most positive), as possible dielectric layers. Experimentally proved that the peak voltage remained at almost the same value, but more negative materials exhibited higher current outputs (Supplementary Material Figure S8 and Figure 5). The average peak *Isc* generated using mica as the dielectric layer was observed at 2.23 mA, with an estimated charge density of about 1.61 C/m^2^. Replacing the mica layer with nylon, PVDF, and PTFE layers resulted in a significant improvement in the average peak *Isc* by approximately 13%, 31%, and 37%, while the transferred charge density increased by 26%, 36%, and 64%, respectively. Interestingly, the maximum current output order matched well with the triboelectric ordering between the materials of the dielectric layers, where the more negative triboelectric materials generated higher current outputs. This increasing trend could be attributed to the increase in the number of triboelectric charges generated by the CE of the SLITF and the dielectric layer. The electropositive SLITF produced more triboelectric charges during CE with a higher negative triboelectric material, resulting in higher current outputs.

### 3.4. Demonstration of the Application of SLITF-TEG

To demonstrate the great potential application of SLITF-TEG as an advanced mechanical energy harvester, we compared its output performance with various cellulose-based nanogenerators and DC mechanical energy harvesters [12,34,61,62,63,64,65,66,67,68,69,70,71,72] (see Supplementary Material Table S1). For instance, Bai et al. [34] developed a high-output CP/LTV-TENG that can produce a peak *Voc* of 478 V and a current density (*Jsc*) of 0.063 A/m^2^. Chen et al. [12] reported a low-cost and efficient DC F-TENG that can generate DC energy with a peak *Voc* of 4500 V and a *Jsc* of 0.0084 A/m^2^. Moreover, Zheng et al. [61] designed high-performance flexible piezoelectric nanogenerators using a flexible porous CNF/PDMS aerogel film that can produce a *Voc* of ~60 V and a *Jsc* of ~0.0505 A/m^2^. Although SLITF-TEG shows a high current output density, the voltage output is relatively low, around 0.5 V, compared to the reported studies. In regard to enhancing the maximum voltage and current outputs, multiple unit cells of SLITF-TEG could be connected in series or parallel and synchronize their outputs. 

**Figure 6:**
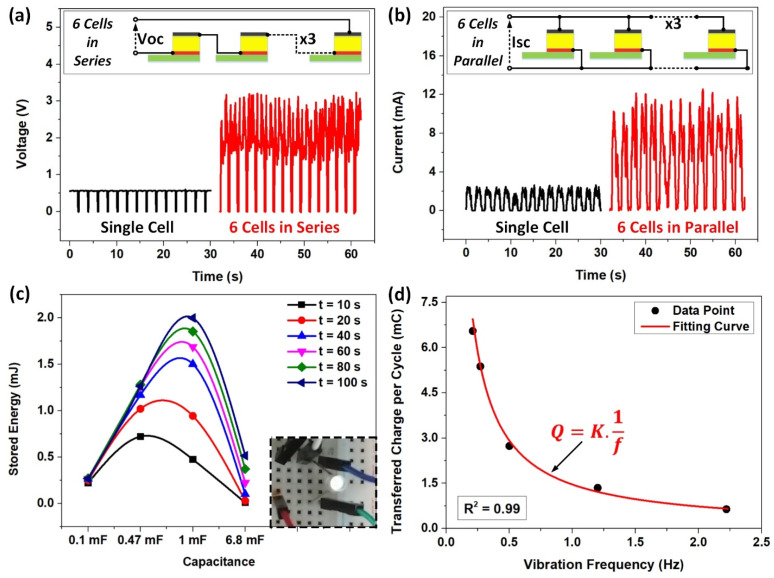
Demonstration of application of SLITF-TEG. (**a**) Enhancing instantaneous voltage by connecting six-unit cells in series. (**b**) Enhancing instantaneous current by connecting six-unit cells in parallel. (**c**) Stored energy concerning the load capacitance for various charging times. Inset image: a light-emitting diode powered by six-unit cells in series. (**d**) Inverse regression analysis between the transferred charge and the frequency of the reciprocating motion.

In this study, SLITF was fabricated by absorbing 2.5 mL water into a cellulose foam with the dimension of 2 cm × 3.5 cm × 0.5 cm (0.37 g-mass). The corresponding electrical responses are reported in Figure 6a,b, which prove that a single cell of SLITF-TEG can produce a maximum instantaneous *Voc* of 0.55 V and *Isc* of 2.5 mA, corresponding to a current density of 3.57 A/m^2^. Intriguingly, six-unit cells connecting in parallel could produce a peak current of about 12.5 mA, and a voltage of approximately 3.2 V could be obtained by six-unit cells connecting in series. Remarkably, the ability to generate DC outputs enables this device to directly charge an energy storage unit, such as a capacitor, without requiring any rectifier. The charging behaviors of different capacitors are presented in Supplementary Material Figure S9. Moreover, the curves of stored energy concerning the load capacitance for various charging times presented in Figure 6c declare the highest stored energy is obtained at 2 mJ in the 1 mF capacitor after a charging time of ~100 s, suggesting that the optimal capacitance is 1 mF. This amount of energy is sufficiently high for directly powering many electronic devices. We experimentally clarified by powering a commercial light-emitting diode directly from six-unit cells of SLITF-TEG connecting in series (inset image of Figure 6c and Supplementary Material Video S4). This proves the promising potential of SLITF-TEG as a power source to run electronic devices for various practical applications.

More interestingly, SLITF-TEG not only provides a mechanical-to-electrical conversion concept but also demonstrates a promising potential application as a self-powered sensor itself based on the dependence of transferred charges during one movement cycle (*Q_C_*) and the vibration frequency (*f*). To validate the sensing capability of the device, we drove the reciprocating motion at various frequencies in a range from 0.21 to 2.22 Hz. The experimental results are presented and discussed above (Figure 4a,b), where the value of *Q_C_* decreases from 6.55 to 0.64 mC/cycle when the applied vibration frequency increases from 0.21 to 2.22 Hz, respectively. After analyzing the data and fitting a curve, we found a strong inversely proportional relationship between the transferred charge during one period of motion and the vibration frequency. The fitting curve can be described by a simple inverse function Equation (6)

(6) $$Q_{C}=K\cdot \frac{1}{f}$$

where *K* is the constant of proportionality, as presented in Figure 6d. The data followed an inverse power function with a constant *K* of 1.4587 and a high coefficient of determination *R*^2^ of 0.99, indicating a high level of accuracy and the potential application of this function in determining the vibration frequency and then sliding velocity.

## 4. Conclusions

In summary, the development of the solid–liquid interface-treated foam-based triboelectric generator (SLITF-TEG) was demonstrated as an effective method to convert mechanical energy into DC power. The SLITF-TEG utilizes cellulose-water interface-treated foam as an active material and generates electricity through the combination of CE due to the sliding friction and the charging effect between two electrodes with different work functions. The study systematically examined the impacts of working parameters and material selection on the SLITF-TEG ‘s output performance. The results demonstrate that triboelectric material properties significantly affect current output, while voltage output depends on electrode pair materials. Under optimized conditions, a single SLITF-TEG can produce DC power with a maximum instantaneous current density of 3.57 A/m^2^ and an induced voltage of approximately 0.55 V. By connecting multiple unit cells of SLITF-TEG in series and parallel, the maximum voltage and current outputs can be increased, and the device can produce enough energy to power electronic devices in various practical applications. Additionally, the SLITF-TEG exhibits a strong inversely proportional relationship between the transferred charge of one movement cycle and the vibration frequency (*R*^2^ = 0.99). Overall, the SLITF-TEG represents a promising potential as an effective strategy for mechanical energy harvesting and self-powered sensor fabrication. 

## Data Availability

The authors confirm that the data supporting the findings of this study is available within the article.

## Supplementary Materials

The following supporting information can be downloaded at: https://www.mdpi.com/article/10.3390/polym15102392/s1, Figure S1: Experimental configuration of the SLITF-TEG; Figure S2: Electrical responses of the SLITF-TEG. The current outputs are measured under (a) the short-circuit condition and (b) various load resistances in the external circuit (10 Ω, 150 Ω, and 1000 Ω); Figure S3: (a) Schematic of the contact area and short-circuit current of the SLITF-TEG. (b) Demonstration of the consistency between the electrical responses and the working mechanism of the SLITF-TEG; Figure S4: The comparison of (a) open-circuit voltage and (b) short-circuit current of the SLITF-TEG in the stationary and sliding modes; Figure S5: (a) The open-circuit voltage and (b) short-circuit current of the SLITF-TEG under different vibration frequencies; Figure S6: (a) The open-circuit voltage and (b) short-circuit current of the SS-TEG using a dry cellulose foam; Figure S7: (a) The open-circuit voltage and short-circuit current of an electrochemical cell. (b) The comparison in output performances of SS-TEG, EC, and SLITF-TEG; Figure S8: (a) The open-circuit voltage and (b) short-circuit current of the SLITF-TEG using different materials of the dielectric layer; Figure S9: (a) Charging behaviors of six-unit cells of SLITF-TEG at different load capacitances. (b) Stored energy-time and stored power-time relationships for a fixed capacitor of 1 mF; Table S1: Peak voltage and current density of various mechanical energy harvesters; Video S1: Measurement of the open-circuit voltage; Video S2: Measurement of the short-circuit current; Video S3: SLITF-TEG directly charges a 6.8 mF capacitor; Video S4: Six-unit cells of SLITF-TEG directly power a light-emitting diode.
