# Supplementary material for: Enhancing the Performance of Triboelectric Generator: A Novel Approach Using Solid–Liquid Interface-Treated Foam and Metal Contacts
Source: Polymers (Basel). 2023 May 20;15(10):2392. doi: 10.3390/polym15102392 (PMC10220941; doi:10.3390/polym15102392)
Supplement: Supplementary file 1 [file polymers-15-02392-s001.zip › polymers-2336450-supplementary.pdf]

polymers

MDPI

Supplementary Material

Enhancing the Performance of Triboelectric Generator: A Novel Approach Using Solid-Liquid Interface-Treated Foam and Metal Contacts

Quang Tan Nguyen [1], Duy Linh Vu [2], Chau Duy Le [1] and Kyoung Kwan Ahn [2,*]

[1] Graduate School of Mechanical Engineering, University of Ulsan, 93, Daehak-ro, Nam-gu, Ulsan 44610, Republic of Korea
[2] School of Mechanical Engineering, University of Ulsan, 93, Daehak-ro, Nam-gu, Ulsan 44610, Republic of Korea
* Correspondence: kkahn@ulsan.ac.kr

Table S1. Peak voltage and current density of various mechanical energy harvesters

Types	Active materials	Size	Voltage (V)	Current density (A/m2)	Refs
Piezoelectric Generator	C/PDMS/AuNPs	3.5 cm x 8.5 cm	6 V	2.35.10-4	[62]
	CMC/PDMS/CNT	3.3 cm x 2.5 cm	30	6.06.10-4	[63]
	Cellulose/SbSI	1 cm x 1 cm	0.024	2.10-4	[64]
	TOCN/PDMS	1 cm x 2 cm	60	5.05.10-2	[61]
	MoS2/PVDF	3 cm x 3 cm	50	3.33.10-5	[65]
	ZnO nanowire array	~2 mm2	10-3	7.5.10-5	[66]
	ZnO nanosheets/Zn: Al layered double hydroxide layer	6 mm2	0.38	0.217	[67]
Triboelectric Generator	Treated wood	4.5 cm x 4.5 cm	81	8.89.10-4	[68]
	PCL/GO	4 cm x 4 cm	120	2.5.10-3	[69]
	Ppy-MWCNT	6.25 cm2	196.8	5.04.10-2	[70]
	CA/PEI/LTV	5 cm x 5 cm	478	6.30.10-2	[34]
	PA/nylon-66	6.8 cm x 7 cm	4500	8.40.10-3	[12]
	FEP/Cu/Steel/Acrylic/ABS	7.5 cm x 7 cm	149.5	4.11.10-3	[71]
	PTFE/Acrylic	10 cm2	33	0.065	[72]
Our generator	Cellulose/Water/Al/Cu	2 cm x 3.5 cm	~0.55	3.57	This work

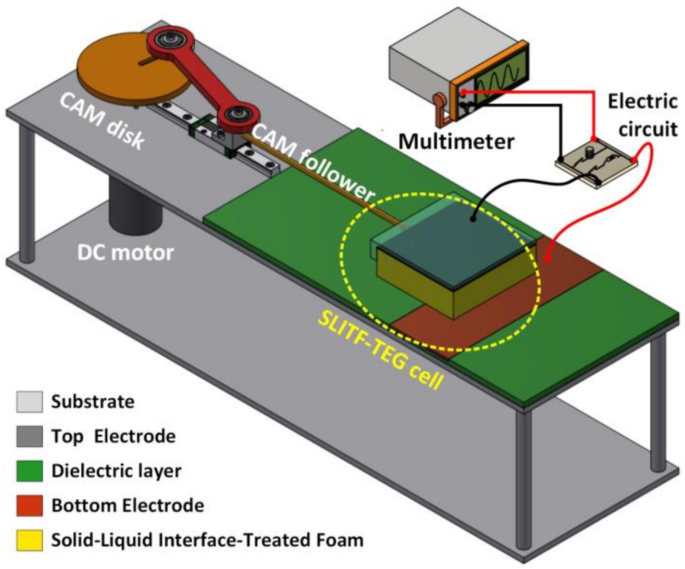

Figure S1. Experimental configuration of the SLITF-TEG

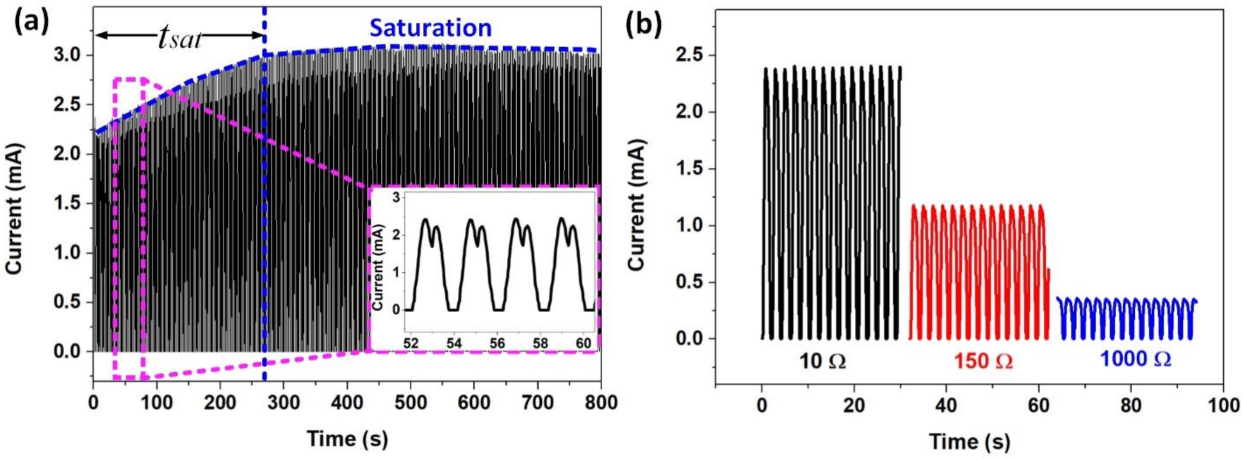

Figure S2. Electrical responses of the SLITF-TEG. The current outputs are measured under (a) the short-circuit condition and (b) various load resistances in the external circuit (10 Ω, 150 Ω, and 1000 Ω).

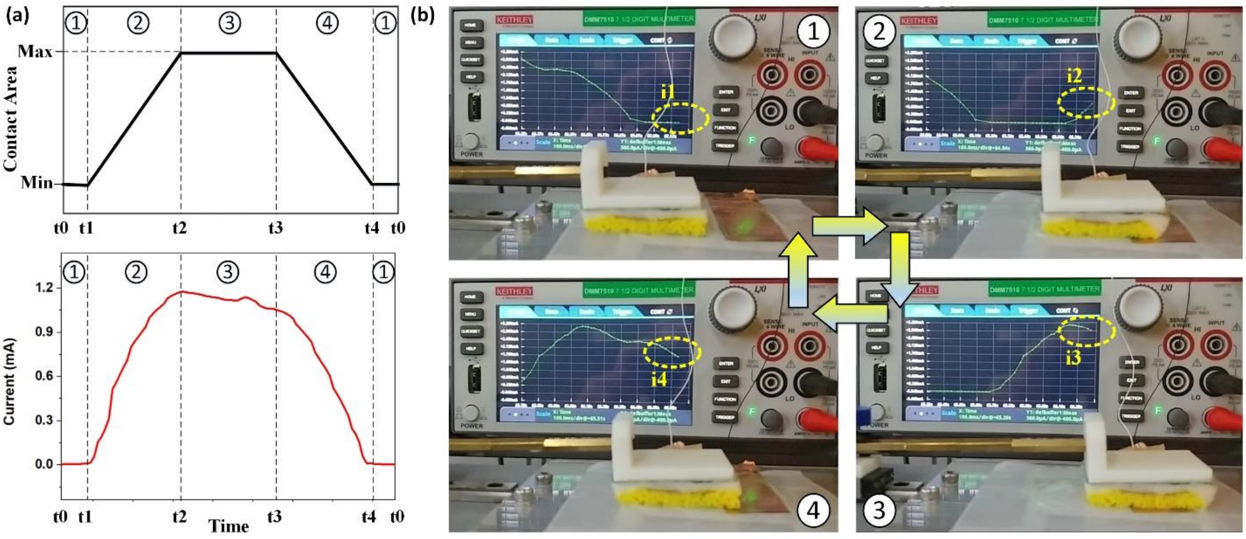

Figure S3. (a) Schematic of the contact area and short-circuit current of the SLITF-TEG. (b) Demonstration of the consistency between the electrical responses and the working mechanism of the SLITF-TEG.

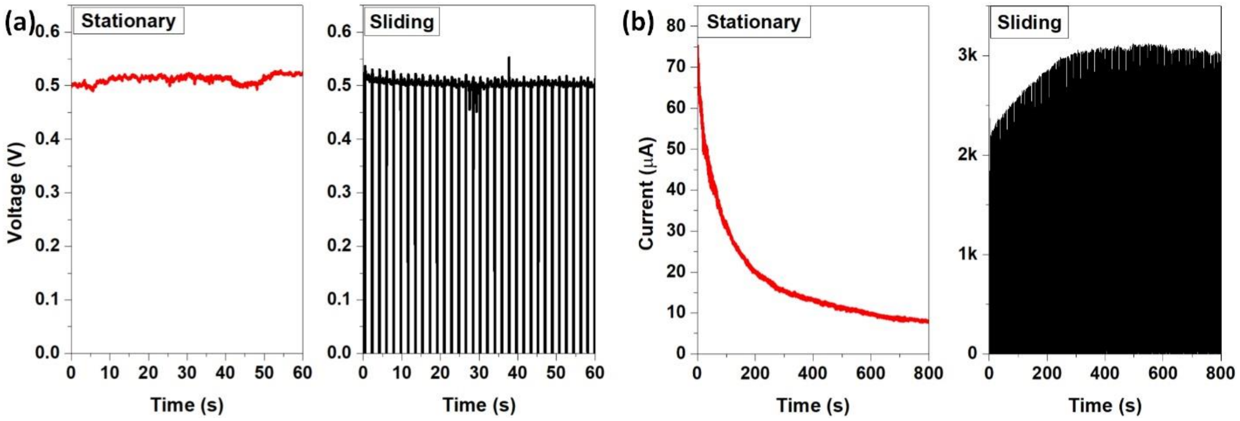

Figure S4. The comparison of (a) open-circuit voltage and (b) short-circuit current of the SLITF-TEG in the stationary and sliding modes.

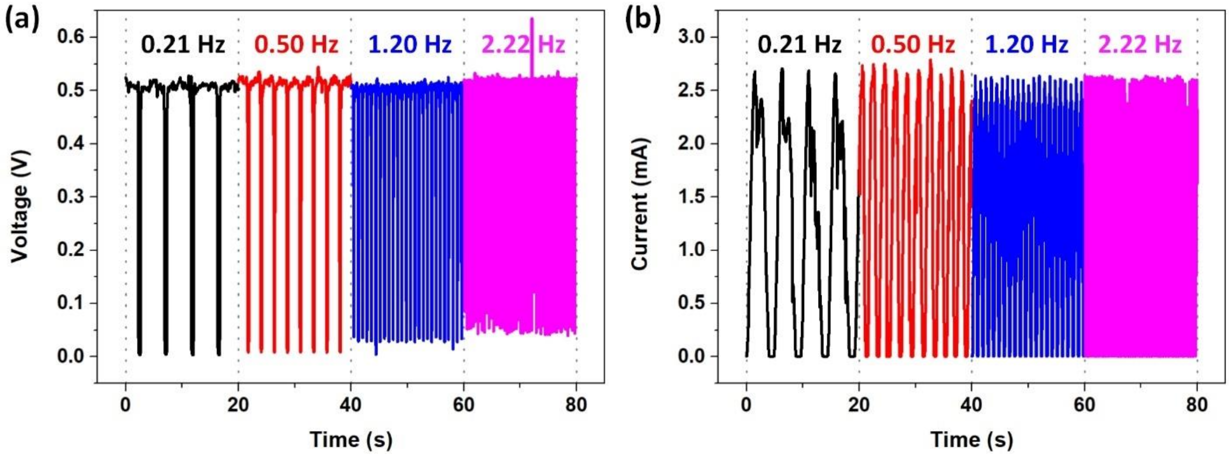

Figure S5. (a) The open-circuit voltage and (b) short-circuit current of the SLITF-TEG under different vibration frequencies.

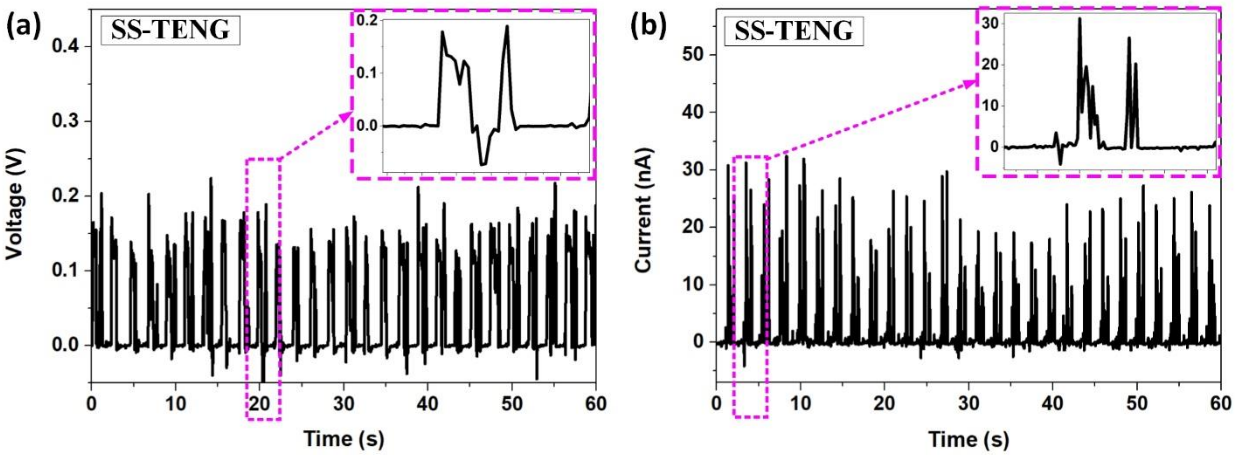

Figure S6. (a) The open-circuit voltage and (b) short-circuit current of the SS-TEG using a dry cellulose foam.

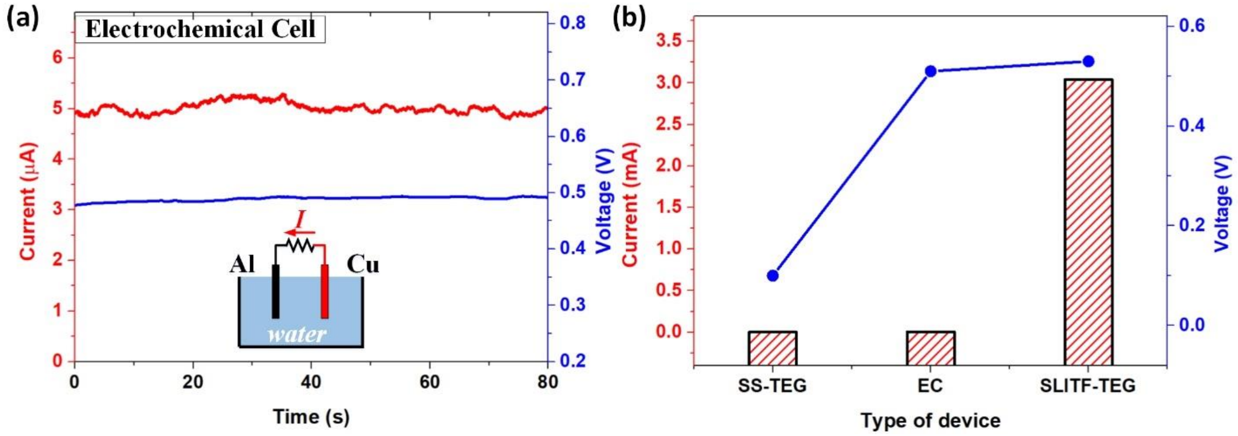

Figure S7. (a) The open-circuit voltage and short-circuit current of an electrochemical cell. (b) The comparison in output performances of SS-TEG, EC, and SLITF-TEG.

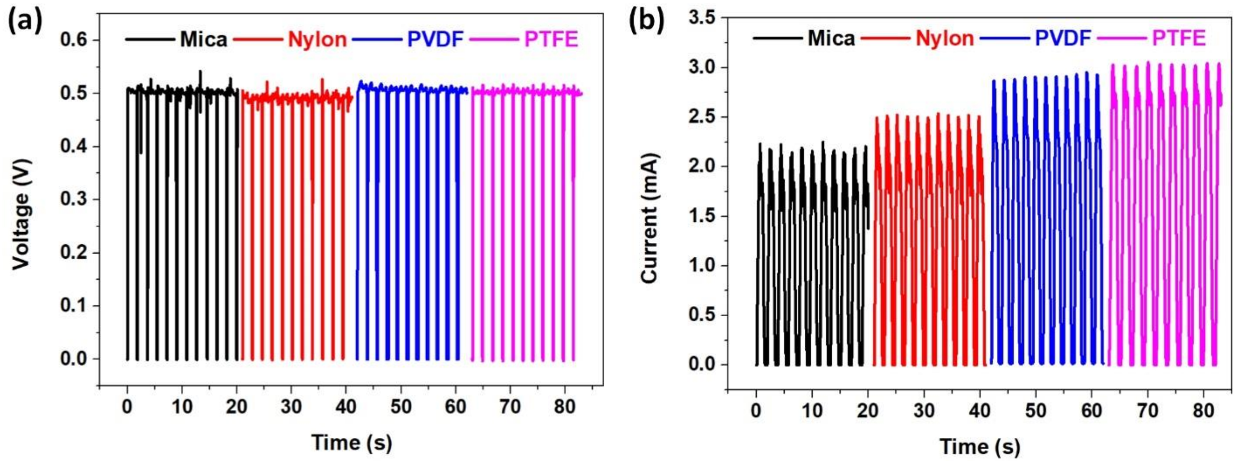

Figure S8. (a) The open-circuit voltage and (b) short-circuit current of the SLITF-TEG using different materials of the dielectric layer.

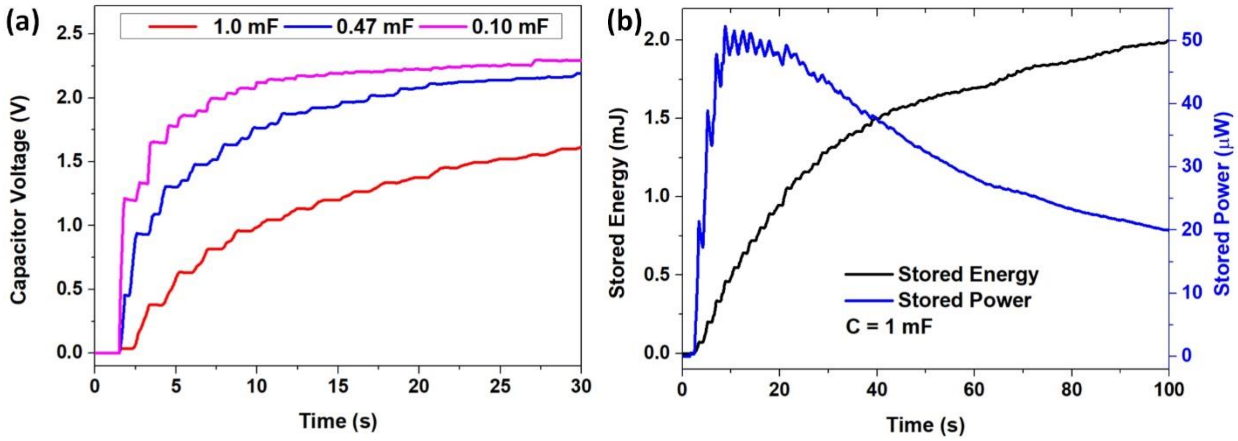

Figure S9. (a) Charging behaviors of six-unit cells of SLITF-TEG at different load capacitances. (b) Stored energy-time and stored power-time relationships for a fixed capacitor of 1 mF.

Video S1: Measurement of the open-circuit voltage

Video S2: Measurement of the short-circuit current

Video S3: SLITF-TEG directly charges a 6.8 mF capacitor

Video S4: Six-unit cells of SLITF-TEG directly power a light-emitting diode
